# Genetic Surveillance of Five SARS-CoV-2 Clinical Samples in Henan Province Using Nanopore Sequencing

**DOI:** 10.3389/fimmu.2022.814806

**Published:** 2022-04-04

**Authors:** Yanan Wang, Duo Chen, Chaofeng Zhu, Zhenhua Zhao, Shanshan Gao, Jianjun Gou, Yongjun Guo, Xiangdong Kong

**Affiliations:** ^1^Genetic and Prenatal Diagnosis Center, the First Affiliated Hospital of Zhengzhou University, Zhengzhou, China; ^2^Department of Clinical Laboratory, the First Affiliated Hospital of Zhengzhou University, Zhengzhou, China; ^3^Department of Pathology, Henan Academy of Medical Sciences, Zhengzhou, China

**Keywords:** SARS-CoV-2, nanopore sequencing, multiplex PCR, nucleotide mutation, phylogenetic analysis, sequencing coverage, virus surveillance, Zhengzhou

## Abstract

Severe acute respiratory syndrome coronavirus 2 (SARS-CoV-2) has rapidly spread and poses a major threat to public health worldwide. The whole genome sequencing plays a crucial role in virus surveillance and evolutionary analysis. In this study, five genome sequences of SARS-CoV-2 were obtained from nasopharyngeal swab samples from Zhengzhou, China. Following RNA extraction and cDNA synthesis, multiplex PCR was performed with two primer pools to produce the overlapped amplicons of ~1,200 bp. The viral genomes were obtained with 96% coverage using nanopore sequencing. Forty-five missense nucleotide mutations were identified; out of these, 5 mutations located at *Nsp2*, *Nsp3*, *Nsp14*, and *ORF10* genes occurred with a <0.1% frequency in the global dataset. On the basis of mutation profiles, five genomes were clustered into two sublineages (B.1.617.2 and AY.31) or subclades (21A and 21I). The phylogenetic analysis of viral genomes from several regions of China and Myanmar revealed that five patients had different viral transmission chains. Taken together, we established a nanopore sequencing platform for genetic surveillance of SARS-CoV-2 and identified the variants circulating in Zhengzhou during August 2021. Our study provided crucial support for government policymaking and prevention and control of COVID-19.

## Introduction

Coronavirus disease 2019 (COVID-19) is a kind of pneumonia caused by SARS-CoV-2. As of November 7, 2021, 250 million people have been infected and more than 5 million people have died. The disease poses a major threat to the public’s physical and mental health around the world. At present, there are no specific drugs that can eradicate COVID-19, and vaccine is the most effective means of preventing COVID-19. There are 7.2 billion vaccine doses administered worldwide, which is a great economic burden to governments (https://coronavirus.jhu.edu/map). However, the effectiveness of vaccines is often compromised by SARS-CoV-2 mutations (1, 2). SARS-CoV-2 is a positive, single-stranded RNA virus with approximately 30-kb genome (3). Compared to DNA virus, it usually leads to higher genetic variability and more rapid evolution (4). Up to now, there have been nine variants according to the nomenclature systems recommended by the World Health Organization (WHO), including Alpha, Beta, Gamma, and Delta. Meanwhile, every variant contains a number of subtypes. The rapid evolution is a great challenge for the genetic surveillance and prevention of COVID-19. Our understanding of SARS-CoV-2 is gradually deepening, mainly owing to the acquisition of the whole genome. Since the first SARS-CoV-2 genome (Wuhan-Hu-1, GenBank accession: MN908947.3) was released (5), different laboratories have submitted over 5 million genome sequences to GISAID, and the data are still constantly updated. Big data will enable us to keep abreast of global virus mutations. Simultaneously, the evolutionary history of a virus can be analyzed over time. More importantly, identification of virus mutation has key effects on vaccine development (6, 7). In the routine reverse transcription quantitative polymerase chain reaction (RT-PCR) diagnosis, false-negative results often happen (8). Li et al. reported a confirmed case of SARS-CoV-2 but with a negative result *via* routine RT-PCR. This case was finally diagnosed using nanopore sequencing; furthermore, they revealed that nucleotide mutations of open reading frame (*ORF*) and nucleocapsid protein (*N*) genes led to the false-positive result. This case highlighted the importance of whole genome sequencing from another perspective (9).

The main strategies used to obtain the whole genome include direct metagenomic sequencing (10), target enrichment sequencing, and PCR amplicon sequencing. The first method is inapplicable to known pathogens because of high cost and incomplete sequencing coverage (11). On the contrary, it is the first choice when facing an unknown pathogen. The reference genome Wuhan-Hu-1 was sequenced using this method. The second method uses virus-specific nucleotide probes for hybridization capture, thus increasing the cost of library preparation. PCR-based enrichment is the most common method for sequencing SARS-CoV-2 genome from clinical samples. Multiple SARS-CoV-2 outbreaks have been identified rapidly by combining PCR amplicons with nanopore sequencing (12–16). Moreover, studies have shown that nanopore sequencing is highly accurate by treating the same sample with next-generation sequencing (17, 18).

In August 2021, a new small-scale outbreak of COVID-19 occurred in Zhengzhou, Henan province. It has been more than a year since the last outbreak, and the emerging SARS-CoV-2 needs to be re-identified. Based on the above background, the aim of our study is to determine the virus strains, track the virus origin, and provide technical support for the government decision-making. In addition, the effects of mutational sites on SARS-CoV-2 structure and function are also evaluated.

## Materials and Methods

### Sample Information

Zhengzhou First People’s Hospital was the designated treatment hospital for SARS-CoV-2-positive patients from August 2021. Nasopharyngeal swabs from five patients admitted to hospital between August and October 2021 were collected and stored in a tube with 3 ml of viral preservation liquid. Samples were incubated at 56°C for 30 min for inactivation.

### Diagnostic RT-qPCR

In order to ensure the integrity of the RNA, viral RNA was extracted as soon as possible upon completion of collection. Following the manufacturer’s instructions (QIAamp Viral RNA Mini Kit, Qiagen), the entire process was performed in the biosafety cabinet. Complementary DNA (cDNA) was synthesized using high-fidelity enzymes. A prepared mix on ice was: random hexamers 1 μl, dNTP 1 μl, template RNA 11 μl, RT buffer 4 μl, dithiothreitol 1 μl, ribonuclease inhibitor 1 μl, and reverse transcriptase 1 μl. The final reaction volume was 20 μl, and the mix was incubated at 42°C for 50 min and at 70°C for 10 min. Set up the RT-qPCR reaction as follows: nucleic acid amplification reaction mix 7.5 μl, enzyme mix 5 μl, primer and probe mix 4 μl, and RNA-free H_2_O 3.5 μl. Virus-like particle containing ORF1ab, N, and RNaseP served as the positive control. Add patient sample, positive or blank control 5 μl to get the final volume 25 μl (JC10223-1N, bioPerfectus technologies). Set up the following cycling conditions: 1 min at 97°C, then 45 cycles of 97°C for 5 s and 58°C for 30 s. RT-qPCR was performed with the Roche LightCycler 480 II PCR system.

### Multiplex PCR for Viral Genome

To amplify the viral whole genome, multiplex PCR using primer pool 1 and pool 2 (BK-WCoV024 II, BAIYITECH) was performed to obtain the tiled amplicons with an overlap of ~100 bp. Mix the following components in a tube: cDNA 2.5 μl, hotstart hifi mix 12.5 μl, primer pool 1/pool 2 4 μl, and nuclease-free water 6 μl. Set up the following PCR program: 30 s at 98°C, then 25–35 cycles of 98°C for 15 s and 65°C for 5 min. The cycle number was determined based on cycle threshold (Ct) value. Pool 1 and pool 2 PCR products were mixed and purified with Agencourt AMPure XP magnetic beads (Beckman Coulter). Before the downstream reaction, PCR products were quantified with a Qubit 2.0 fluorometer following the specifications of Qubit dsDNA high-sensitivity (HS) assay kit.

### Library Construction and Nanopore Sequencing

PCR products were treated with a ligation kit (SQK-LSK109, Oxford Nanopore Technologies) and a barcoding kit (EXP-NBD114, Oxford Nanopore Technologies). NEBNext FFPE DNA Repair Mix and NEBNext Ultra II End repair/Da-tailing module reagents were prepared according to the manufacturer’s instructions. Barcoded DNA (1 μl) was quantified for the subsequent adapter ligation. DNA library was prepared with 400 ng of barcoded DNA and purified once more prior to sequencing. MinION MK1B sequencer (Oxford Nanopore Technologies) was used for whole genome sequencing with R9.4.1 flow cells. It was important that the flow cell priming should be completed before loading the DNA library. MinKNOW software was used to start the sequencing run for 12 h.

### Bioinformatic Analysis

First, the raw nanopore electrical signal (.fast5) was converted to base sequence (.fastq) through high-accuracy basecalling with MinKNOW. We chose the offline basecalling mode after the sequencing run was stopped. Reads with mean_qscore_template more than 10 were defined as “pass” and demultiplexed using Guppy v5.0.7. A maximum of 4,000 entries were put into a fastq directory. Afterwards, reads with a sequencing length of >1,400 bp or <800 bp were removed. The clean data were aligned to the reference genome Wuhan-hu-1 using Minimap2 v2.22, and then primers were trimmed from reads following the align_trim.py of Artic workflow. In order to identify single nucleotide variations (SNVs), variant calling was performed using medaka v.1.4.3 and longshot v0.4.1 according to the protocol recommended by Artic network. Set up the filter conditions as follows: flag=0 or 16, mapQ ≥ 60, DP ≥ 20. Consensus sequencing was generated by bcftools v1.14. Sequencing coverage was quantified with mosdepth v0.3.2 and visualized using R. If sequencing depth was less than 20×, the base was labeled as N. For heterozygous sites, the bases with higher sequencing depth were selected. In general, our bioinformatic workflow contained basecalling, demultiplexing, alignment with the reference genome, primer trimming, variant calling, and building the consensus sequence.

### Phylogenetic Analysis

Five sequencing genomes were uploaded to the online Pangolin, and lineage classification was performed with Phylogenetic Assignment of Named Global Outbreak LINeages. The cumulative prevalence of each lineage at every location was recorded by Mutation Tracker. Meanwhile, clade analysis was done using Nextclade tool, and the phylogenetic tree was performed and visualized with Prism GraphPad 9. To determine the most likely source of five SARS-CoV-2 samples, 189 sequences from other regions of China and Myanmar were downloaded from GISAID, and a multiple sequence alignment of all the above 194 sequences was conducted using Mafft v7.490. The phylogenetic tree was generated using FastTree with -gtr parameter, and further visualized and annotated using the R package ggtree V2.4.2.

### Software and Database

GraphPad Prism 9 was used to perform the simple linear regression. In this study, the following database was used. The sequencing data of five patient samples were deposited in GISAID (https://www.gisaid.org/), and accession IDs are listed in **Table 1**. The global SNV statistics of SARS-CoV-2 were investigated using GISAID and NCBI. The lineage of genome was analyzed using the online website pangolin (https://pangolin.cog-uk.io/), and Nextclade (https://clades.nextstrain.org/) was used for tracking the clade.

**Table 1:** Patient information and routine RT-qPCR results.

Sample	Patient 1	Patient 2	Patient 3	Patient 4	Patient 5
Sex	Female	Male	Male	Female	Male
ORF1ab	18	20	28	26	34
N	16	20	27	26	32
Coverage ≥100	99.58%	99.60%	99.59%	99.60%	97.08%
Coverage ≥1,000	94.24%	79.20%	79.51%	83.99%	72.49%
Coverage	99.61%	99.64%	99.63%	99.63%	99.62%
Depth	17,631×	14,137×	14,699×	16,724×	12,251×
Total bases	527Mb	423Mb	440Mb	500Mb	366Mb
GISAID accession ID	EPI_ISL_5462229	EPI_ISL_5462235	EPI_ISL_5462243	EPI_ISL_5462244	EPI_ISL_5462252
N	115	108	111	112	112
Lineage	B.1.617.2	B.1.617.2	AY.31	AY.31	AY.31

## Results

### Five Patients Were Diagnosed by Routine RT-qPCR Test

**Figure 1:**
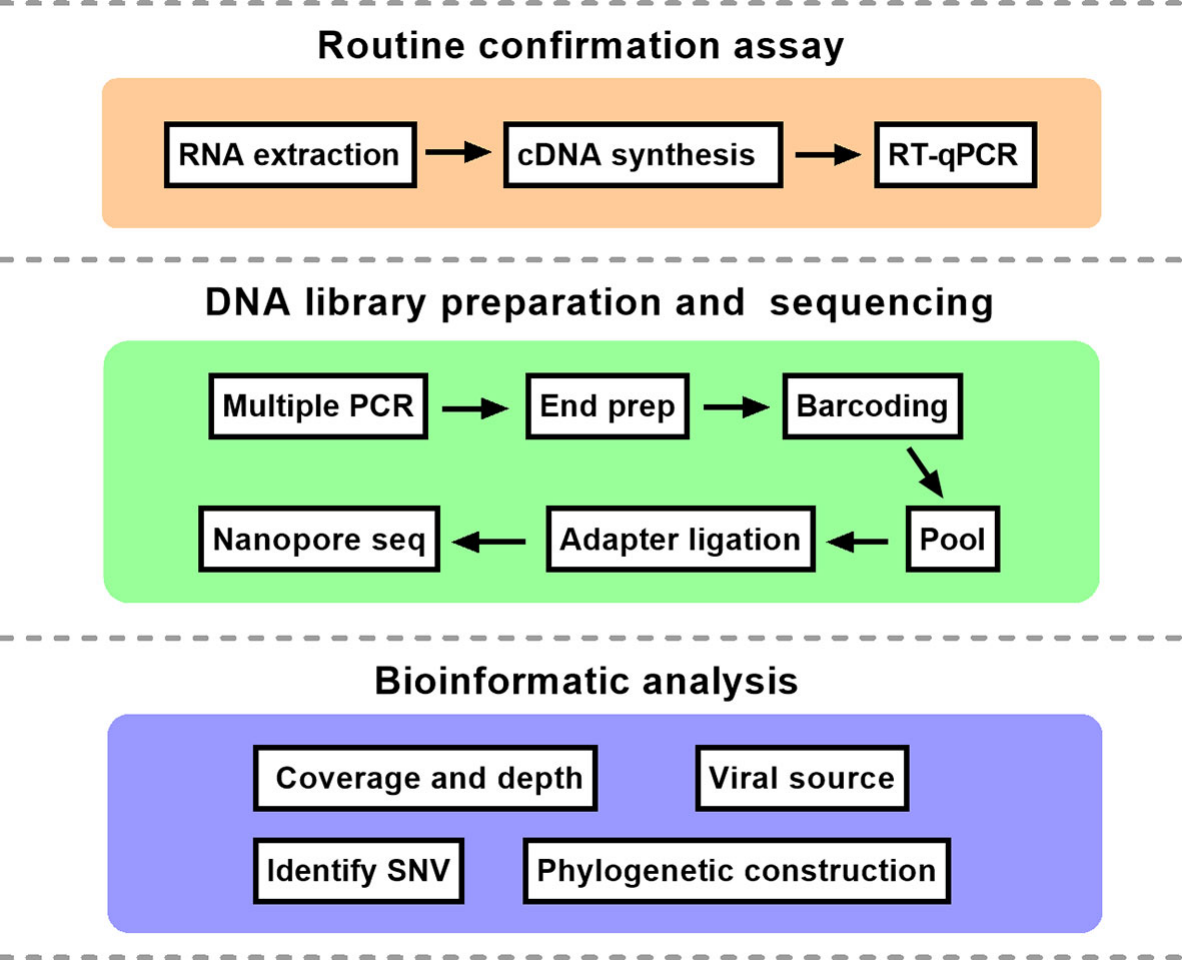
The schematic overview of nanopore sequencing based on multiplex PCR. The protocol mainly consisted of three parts. RT-qPCR, reverse transcription quantitative polymerase chain reaction; SNV, single nucleotide variation.

The comprehensive workflow is described in **Figure 1**. cDNA was synthesized after viral RNA was extracted, and then cDNA was subjected to RT-qPCR with two pairs of specific primers for *ORF1ab* and *N* genes. A Ct value of less than 37 was defined as positive, and a Ct value of 37 or more was deemed negative. If only one gene was measured with a Ct value below 37, the corresponding sample should be retested. In our study, Ct values of five patient samples ranged from 16 to 34 as shown in **Table 1**. For the same sample, there was little difference in Ct value between *ORF1ab* and *N* genes.

### Five SARS-CoV-2 Genomes Were Obtained With High-Sequencing Coverages and Depths

**Figure 2:**
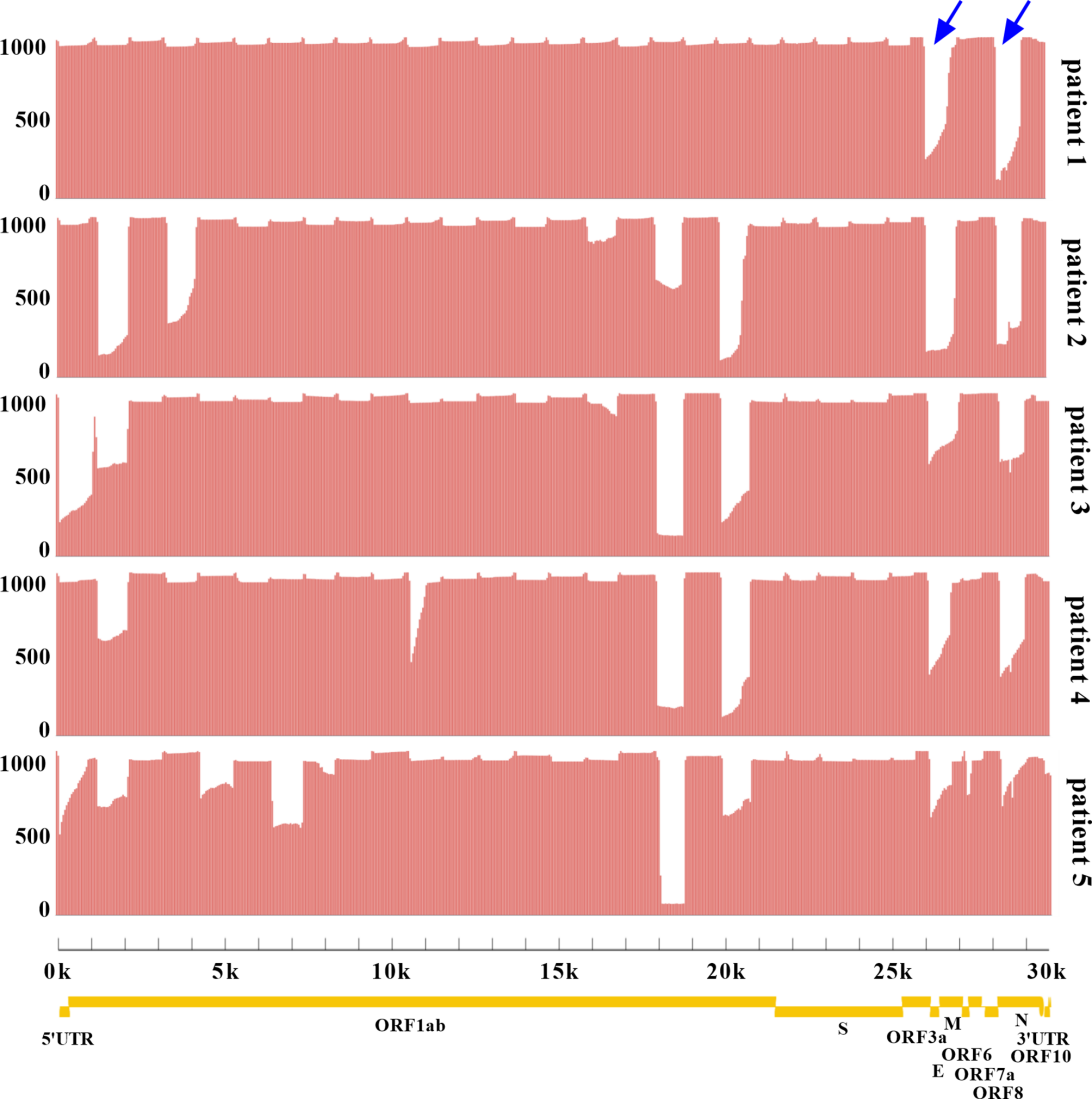
Analysis of sequencing coverage and depth for each SARS-CoV-2 sample. The multiplex PCR products were sequenced with R.9.4.1 flow cell. The horizontal axis represented the physical location of SARS-CoV-2 genome from 5’UTR-3’UTR, and the vertical axis represented the sequencing depth of each physical location. The whole genome coverage and depth of each amplicon were shown. Yellow indicated the genes of SARS-CoV-2 encoding structural proteins (S, spike; E, envelope; M, membrane; N, nucleocapsid) and non-structural proteins (ORF1ab, ORF3a, ORF6, ORF7a, ORF7b, ORF8, and ORF10). UTR. untranslated region; ORF, open reading frame. Blue arrows highlighted the amplicons with low sequencing depth.

Two sets of primers were designed for amplifying the non-adjacent regions and covering the whole genome of SARS-CoV-2. Pool 1 contained 15 primer pairs in odd positions and pool 2 had 14 primer pairs in even positions. The length of each amplicon was ~1200 bp with an overlapped region of ~100 bp. Amplicons of two pools were mixed to obtain the intact DNA library. After sequencing for 12 h, total bases were obtained with an average of 451.2 megabases (Mb). The coverage of each sample was up to 96% of the whole genome. We found that the remaining 4% bases were uncovered and labeled as Ns (<20×), which were mostly located at the 3’ end of SARS-CoV-2 genome. Only 4 Ns were located at the 5’ end for patient 1. To assess the multiplex PCR, sequencing coverage and depth of each amplicon were quantified and visualized in **Figure 2**. Results indicated that nanopore sequencing covered the whole genome of SARS-CoV-2 with a different sequencing depth. Specifically, the proportion of regions with sequencing depth ≥100× was more than 99% for 5 patient samples. When sequencing depth was ≥1,000×, the proportions were 94.24% for patient 1, 79.20% for patient 2, 79.51% for patient 3, 83.99% for patient 4, and 72.49% for patient 5. In general, a majority of amplicons reached a sequencing depth of ≥1,000×. Meanwhile, we noticed that there was an obvious difference between primer pool 1 and pool 2. As indicated by blue arrows in **Figure 2**, the sequencing depths of some amplicons were much lower than the adjacent regions. These problematic amplicons were obtained with the following primer pairs (2, 4, 18, 20, 26, and 28) of pool 2. Compared to pool 2, there was a better evenness of sequencing depth for pool 1.

### Sequencing Coverage Was Associated With Ct Value

**Figure 3:**
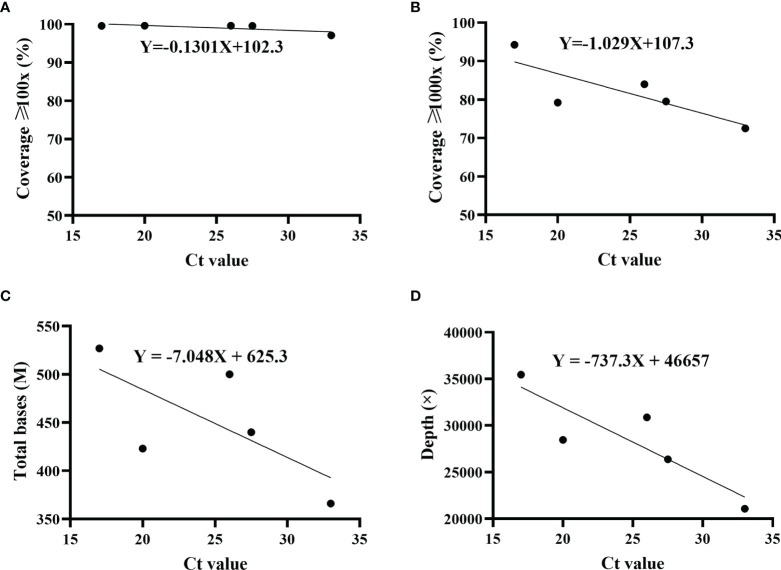
Correlation of sequencing parameters and Ct value. The correlation was analyzed using simple linear regression. Ct values were determined by *ORF1ab* and *N* genes, and the average was displayed. For each set, including sequencing coverage **(A, B)**, ≥ total bases **(C)** and sequencing depth ≥ **(D)** with different Ct values, regression equation was calculated. Patient 1, *R*^2^ = 0.5356; patient 2, *R*^2^ = 0.6551; patient 3, *R*^2^ = 0.4867; patient 4, *R*^2^ = 0.7648.

The proportions of sequencing depth ≥1,000× varied among five patients. Whether the difference was due to Ct value (viral load) was not yet confirmed. Therefore, the correlation of sequencing coverage and Ct value was evaluated. Results indicated that Ct value did not have an effect on the coverage with sequencing depth ≥100× (**Figure 3A**). However, as Ct value increased, the sequencing coverage ≥1,000× decreased gradually (**Figure 3B**). Furthermore, there were a similar trend for total base number and average sequencing depth, which were negatively correlated with the Ct value (**Figures 3C, D**).

### Five Samples Were Divided Into Two Subclades Based on Different Mutation Profiles

**Figure 4:**
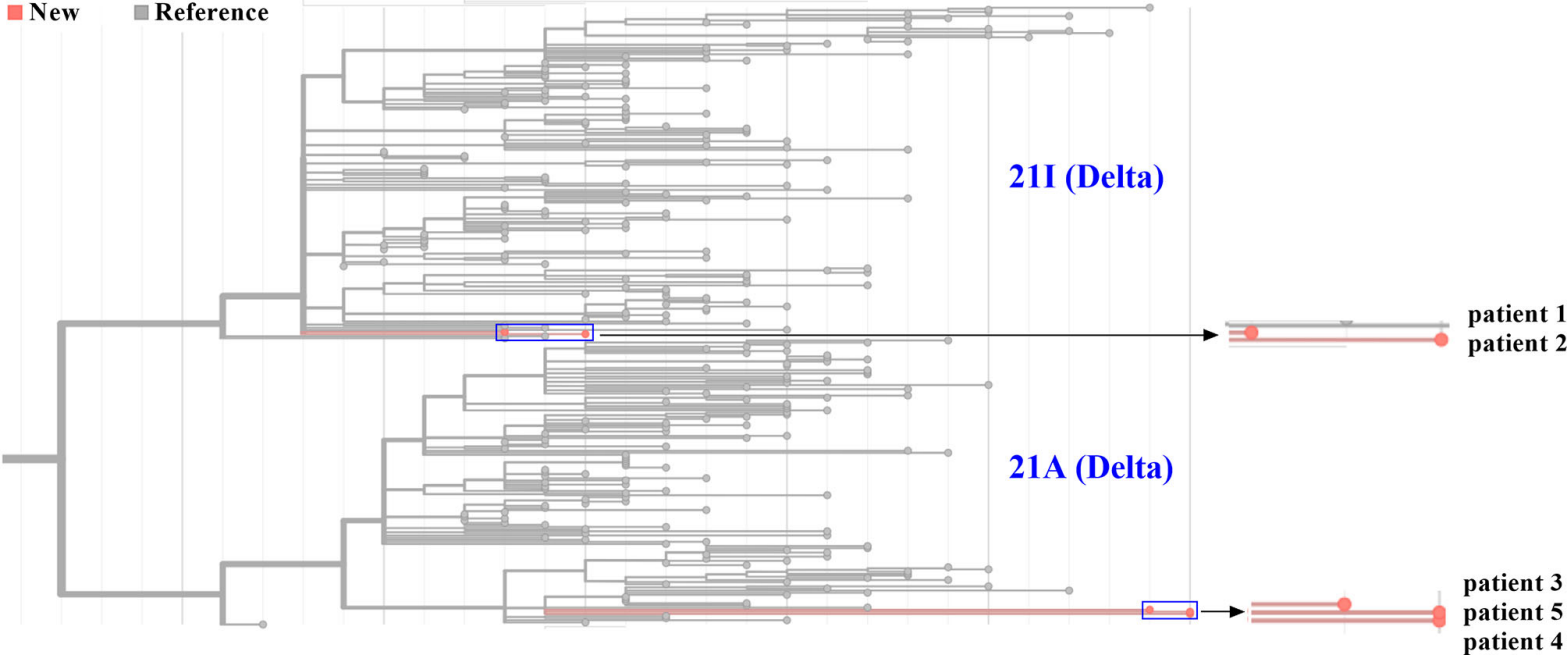
Phylogenetic analysis of five SARS-CoV-2 genomes from Zhengzhou. The phylogenetic tree was generated using Nextclade and visualized using Prism GraphPad 9. Line with red dots indicated our sequences, and line with gray dots indicated the reference sequences. By default, all sequences divided into 21A and 21I subclades in the Nextclade tool were displayed. The horizontal axis represented the number of mutations. The blue rectangle was zoomed in and displayed on the right.

Five genome sequences were uploaded to the online Pangolin COVID-19 lineage assigner. To our surprise, although five samples came from patients infected in the same outbreak, they were assigned to different sublineages. Patients 1 and 2 belonged to the B.1.617.2 sublineage, while three other samples were assigned to the AY.31 sublineage (**Table 1**). B.1.617.2 included all AY sublineages, and the proportion of AY.31 sublineage was less than 4.2%. As of November 2021, the cumulative prevalence of B.1.617.2 sublineage worldwide was 12%. Furthermore, five genome sequences were analyzed using Nextclade. Results revealed that patients 1 and 2 were aligned to 21I clade, and patients 3–5 were aligned to 21A clade based on different mutation profiles. 21I and 21A are the subclades of the Delta variant. The classification result of the above two systems was similar (**Figure 4**). The detailed nucleotide mutations and amino acid changes were investigated and displayed in **Supplementary Table 1**. According to sequencing depth, a total of 65 nucleotide mutations were identified for five samples relative to the reference genome. The numbers of mutations were 33, 35, 47, 48, and 48 for patients 1–5, respectively. AY.31 sublineage produced more nucleotide mutations relative to B.1.617.2. Out of 65 SNVs, 16 were synonymous mutations, and 49 other missense mutations contained the characteristic mutations in B.1.617.2 or AY.31 sublineage. These mutations (p.Leu452Arg, p.Thr478Lys, p.Asp614Gly, p.Ser640Pro, and p.Pro681Arg) with high prevalence were reported a lot, which were often involved in phenotypic changes and affected ligand binding (19–22). They played a vital role in the virus infectivity and mortality rate (23). For the uncommon mutations in our study, the global statistics for these positions were further estimated using GISAID. There were 5 mutations (ORF1ab: p.Ser531Ile, p.Lys1280Asn, p.Met2194Ile, p.Ile6399Val; ORF10: p.Asn25Ser) with <0.1% prevalence. Out of these, p.Ser531Ile only appeared in one sequence of the global dataset. These mutations with lower prevalence were related to *Nsp2*, *Nsp3*, *Nsp14*, and *ORF10* genes. In addition, three mutations were found at the non-coding regions of 5’ end and 3’ end, including n.210G>T, n.241C>T, and n.29742G>T, and their biological effects and epidemiological significance remained unknown.

### Phylogenetic Analysis Revealed Potentially Different Virus Sources for Five Samples

**Figure 5:**
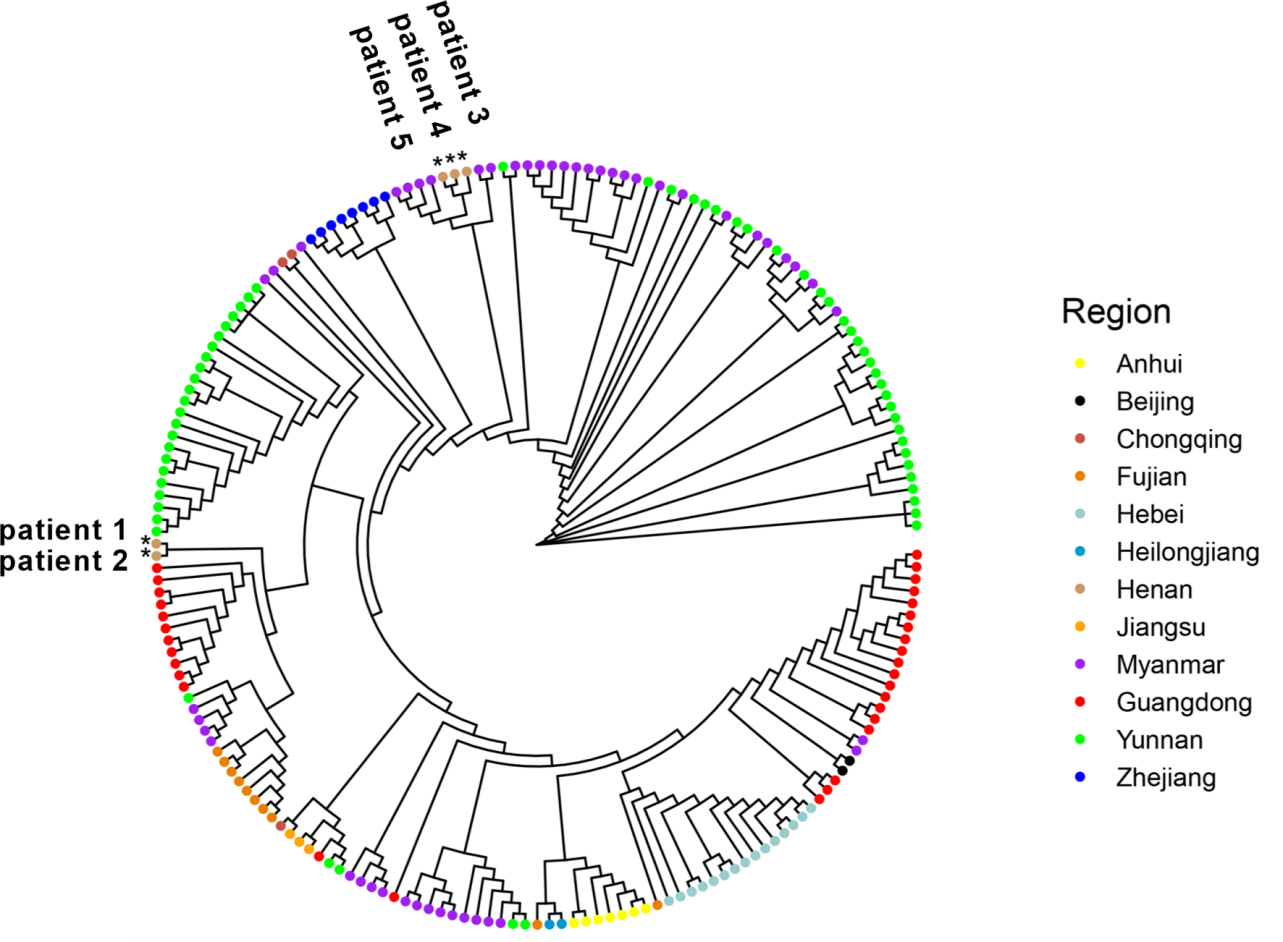
Five genome sequences in this study were aligned with 141 genomes from other provinces of China. In addition, 48 sequences from Myanmar were also added. All sequences were selected from January through November 2021 and downloaded from GISAID. Samples in this study were labelled as *.

Since January 2021, there have been multiple small outbreaks of SARS-CoV-2 in different regions of China. In particular, the epidemic in Henan province came on the heels of a Delta outbreak in Jiangsu province. According to the epidemiological investigation, the initial infected patients (patients zero and one, not patients in this study) were linked to the confirmed cases who had a travel history from Myanmar. One of our goals was to determine the virus source by whole genome alignment. Taking five samples in this study and 189 genomes from other regions of China and Myanmar for phylogenetic reconstruction, results revealed that five samples were mainly grouped into different subclades. Sequences of patients 3–5 were clustered into the ones from Myanmar, and they had a high homology (**Figure 5**). This indicated that the epidemic in Henan province was not associated with that in Jiangsu province, which was in line with official reports. Patients 1 and 2 belonged to a separate subclade. They were indirectly related to the local hospital based on epidemiological data, and genome sequencing showed a moderate homology with other strains from Myanmar, which may indicate a distinct virus transmission chain.

## Discussion

By 2020, the population of Henan province has reached 99.4 million, accounting for 7% of the country’s total population. As the capital of Henan province, Zhengzhou is located in the central region of China, and is an important comprehensive transportation hub in China. The high population density and developed transportation network are huge challenges to the prevention and control of SARS-CoV-2. This outbreak in August 2021 started at a local hospital that was responsible for nucleic acid testing and quarantine of inbound flights. The virus quickly spread to surrounding areas, and local residents with an initial positive screening were admitted to the designated treatment hospital. Five patients were randomly selected in this study. For the routine RT-qPCR assay, we chose *ORF1ab* and *N* genes as the targets. These conserved regions of SARS-CoV-2 genome were usually recommended to design the specific primer and probe in the RT-qPCR. To ensure the accuracy, only double positives were considered as true. Instead of the primer schemes (98 pairs) suggested by artic-ncov2019 (17, 24) (https://artic.network/ncov-2019), the nearly whole genome was enriched by a long-fragment multiple PCR protocol. Using fewer primer pairs was more conducive to reducing the differences among amplicons. Studies have shown that the length of 1,200 bp was the most appropriate relative to other shorter or longer fragments (25). Twenty-nine primer pairs were used for the multiplex PCR. Compared to primer pool 2, we have noted that there was a better coverage evenness for primer pool 1. Primer design needs to be optimized for the following amplicons amplified by pool 2, including amplicons 2, 4, 18, 20, 26, and 28. At these physical locations, the sequencing depths were considerably lower than the average. Similarly, the amplicon dropouts also happened in other studies (25–27). The reason may be that primer mismatch occurred in these locations, and amplicons were affected by mutations in the B.1.617.2 sublineage. In general, compared to previous studies with nanopore sequencing, the sequencing coverage was higher (99%) at ≥100× depth (28–30). As shown in **Figure 3**, the Ct value had an effect on multiple sequencing parameters. Our study indicated that Ct value was negatively correlated with total bases and sequencing depth. This tendency was consistent with the results from a previous study (31). The higher the Ct value, the lower the viral load. For the multiplex PCR, the cycle number should be adjusted according to the Ct value. Simultaneously, DNA products at each step should be accurately quantified to ensure the consistency of barcoding samples.

A nanopore MinION sequencer was applied in our study due to its portability and efficiency. Firstly, it was easy to bring a sequencer into isolated hospitals with strict control or some rural areas with limited resources, thus saving sample turnaround time (32). Secondly, the operation process from virus extraction to bioinformatic analysis was simple and can be completed in a short time, which did not require much technical expertise. Thirdly, multiple SARS-CoV-2 samples can be detected simultaneously in one experiment through barcoding, thus reducing the sequencing cost (33). However, undeniably, the overall error rate of indel and substitution by nanopore sequencing technology is higher (6%–15%) than Illumina short-read sequencing (0.1%–1%) (34, 35). Errors occur randomly without hotspots. The problem can be resolved with enough sequencing depth (13, 36). As mentioned above, our average sequencing depth was 15,088×. Therefore, the error rate can be ignored in our study. Forty-nine missense mutations were identified by deep sequencing for five samples, and five rare mutations were found at *Nsp2* (p.Ser531Ile), *Nsp3* (p.Lys1280Asn and p.Met2194Ile), *Nsp14* (p.Ile6399Val), and *ORF10* (p.Asn25Ser) genes. These genes encoded non-structural proteins. The direct effects of Nsp2 remained not yet clear, and Nsp2 may enhance the viral replication by intervening the autophagy defense response and mitochondrial dysfunction (37, 38). The protein structure and amino acid mutations were rarely reported. Nsp3 is a protein with a papain-like protease domain involved in cleaving the viral polyprotein. In particular, Nsp3, Nsp4, and Nsp6 play a fundamental role in the biogenesis of the double-membrane vesicles (39). Nsp14 is an important protein with exonuclease activity and responsible for error correction during RNA replication. Meanwhile, Nsp14 has methyltransferase activity, which can prevent viral RNA from being recognized by host cells as foreign RNA and thus is degraded (40, 41). A recent study reported that the *ORF10* gene is not a protein-coding gene and is unnecessary for SARS-CoV-2 replication. It is not essential in human *in vitro* or *in vivo* (42). The mutation frequencies of the above nucleotides are low in the global dataset. Therefore, the phenotypic and functional effects of every mutation need to be further evaluated in the presence of experimental models and epidemiological investigation (43). This points out the directions for our future work.

We confirmed the presence of two sublineages by phylogenetic analysis. Five SARS-CoV-2 genomes were the first uploaded genome data from Henan province during this outbreak. The phylogenetic analysis of whole genomes from other regions of China and Myanmar was consistent with epidemiological investigation. The virus of patients 3–5 came from Myanmar. The AY.31 sublineage was closely related to Myanmar. According to the reports of WHO, at the time of designation, almost all AY.31 sequences originated from Myanmar. At present, the cumulative prevalence of the AY.31 sublineage is highest (4%) in Myanmar, and AY.31 is detected in China for the first time in this study. The cumulative prevalence of the B.1.617.2 sublineage is 9% in China, and the high prevalence is over 85% in Fiji and Vietnam. The highest number of total cases is in India, and the B.1.617.2 sublineage has spread to many countries. Phylogenetic analysis revealed that patients 1 and 2 belonged to a separate subclade, which did not have a very high homology with 189 reference genomes including ones from Myanmar. The reason may be the limitation in the number of reference genomes. In addition, there were different strains in Myanmar, and some sequences may not be uploaded to GISAID. Patients 1 and 2 had a distinct virus transmission chain.

Of note, patient 4 was confirmed with a Ct value of 26. Although her viral load was low, she tested positive for a longer period relative to other patients. After other clinical complications were excluded, we hoped to dig up more information by nanopore sequencing. However, no specific mutations were found for patient 4. In the real world, the interaction of virus and host was complicated, and more efforts should be made to elucidate the cause. One of the limitations of this study was the small sample size of patients. It was difficult to draw definite conclusions about the origin and evolution of the virus. Although the sequencing depth was high enough, it will be more precise if the samples can be confirmed using next-generation sequencing. In summary, our results suggest that nanopore sequencing based on multiplex PCR is an effective method to obtain the whole genome of SARS-CoV-2. The timely sharing of SARS-CoV-2 genome is beneficial to understand the virulence and transmissibility and guide local surveillance and prevention.

## Data Availability Statement

The datasets presented in this study can be found in online repositories. The names of the repository/repositories and accession number(s) can be found in the article/**Supplementary Material**.

## Ethics Statement

Written informed consent was obtained from the individual(s) for the publication of any potentially identifiable images or data included in this article.

## Author Contributions

XK, YG and JG conceived and designed this study. RNA extraction, cDNA synthesis, multiplex PCR, and nanopore sequencing were performed by DC, CZ, and SG. YW and ZZ conducted the bioinformatic analysis. YW wrote the manuscript. All authors have discussed the results, revised the manuscript, and agreed to the submission.

## Funding

This work was supported by Key Technology R&D Program for Epidemic Prevention and Control of Henan Province (211100310900).

## Conflict of Interest

The authors declare that the research was conducted in the absence of any commercial or financial relationships that could be construed as a potential conflict of interest.

## Publisher’s Note

All claims expressed in this article are solely those of the authors and do not necessarily represent those of their affiliated organizations, or those of the publisher, the editors and the reviewers. Any product that may be evaluated in this article, or claim that may be made by its manufacturer, is not guaranteed or endorsed by the publisher.
