# Supplementary material for: Genetic Surveillance of Five SARS-CoV-2 Clinical Samples in Henan Province Using Nanopore Sequencing
Source: Front Immunol. 2022 Apr 4;13:814806. doi: 10.3389/fimmu.2022.814806 (PMC9013895; doi:10.3389/fimmu.2022.814806)
Supplement: Supplementary file 1 [file Table_1.docx]

Supplementary Material

**Supplementary Table 1. Summary of SNVs detected in five patient samples from Zhengzhou**

| **Position** | **Mutation type** | **CDS** | **Nucleotide acid change** | **Aminoacid change** | **Patient** |
| --- | --- | --- | --- | --- | --- |
| **210** | **intergenic** | **START-orf1ab** | **n.210G>T** |  | **1,2,3,4,5** |
| **241** | **intergenic** | **START-orf1ab** | **n.241C>T** |  | **1,2,3,4,5** |
| **1267** | **synonymous** | **orf1ab** | **c.1002C>T** | **p.Gly334Gly** | **3,4,5** |
| **1857** | **missense** | **orf1ab** | **c.1592G>T** | **p.Ser531Ile** | **2** |
| **3037** | **synonymous** | **orf1ab** | **c.2772C>T** | **p.Phe924Phe** | **1,2,3,4,5** |
| **3306** | **missense** | **orf1ab** | **c.3041T>C** | **p.Met1014Thr** | **3,4,5** |
| **4099** | **synonymous** | **orf1ab** | **c.3834C>T** | **p.Phe1278Phe** | **3,4,5** |
| **4105** | **missense** | **orf1ab** | **c.3840G>T** | **p.Lys1280Asn** | **1** |
| **4780** | **synonymous** | **orf1ab** | **c.4515C>T** | **p.Ile1505Ile** | **3,4,5** |
| **4927** | **synonymous** | **orf1ab** | **c.4662C>T** | **p.Asp1554Asp** | **3,4,5** |
| **5184** | **missense** | **orf1ab** | **c.4919C>T** | **p.Pro1640Leu** | **1,2,3,4,5** |
| **5584** | **synonymous** | **orf1ab** | **c.5319A>G** | **p.Thr1773Thr** | **1,2** |
| **6539** | **missense** | **orf1ab** | **c.6274C>T** | **p.His2092Tyr** | **3,4,5** |
| **6847** | **missense** | **orf1ab** | **c.6582G>T** | **p.Met2194Ile** | **2** |
| **9891** | **missense** | **orf1ab** | **c.9626C>T** | **p.Ala3209Val** | **1,2,3,4,5** |
| **10319** | **missense** | **orf1ab** | **c.10054C>T** | **p.Leu3352Phe** | **2** |
| **10573** | **synonymous** | **orf1ab** | **c.10308T>C** | **p.Ala3436Ala** | **3,4,5** |
| **11418** | **missense** | **orf1ab** | **c.11153T>C** | **p.Val13718Ala** | **1,2,3,4,5** |
| **11514** | **missense** | **orf1ab** | **c.11249C>T** | **p.Thr3750Ile** | **1,2** |
| **12946** | **synonymous** | **orf1ab** | **c.12681T>C** | **p.Tyr4227Tyr** | **3,4,5** |
| **13019** | **synonymous** | **orf1ab** | **c.12754C>T** | **p.Leu4252Leu** | **1,2** |
| **13072** | **synonymous** | **orf1ab** | **c.12807C>T** | **p.Phe4269Phe** | **1,2** |
| **14262** | **synonymous** | **orf1ab** | **c.13998C>T** | **p.Asp4666Asp** | **3,4,5** |
| **14408** | **missense** | **orf1ab** | **c.14144C>T** | **p.Pro4715Leu** | **1,2,3,4,5** |
| **15451** | **missense** | **orf1ab** | **c.15187G>A** | **p.Gly5063Ser** | **1,2,3,4,5** |
| **16466** | **missense** | **orf1ab** | **c.16202C>T** | **p.Pro5401Leu** | **1,2,3,4,5** |
| **16733** | **missense** | **orf1ab** | **c.16469C>T** | **p.Ser5490Leu** | **5,6,7** |
| **17331** | **missense** | **orf1ab** | **c.17067G>T** | **p.Glu5689Asp** | **3,4,5** |
| **17964** | **missense** | **orf1ab** | **c.17700G>T** | **p.Met5900Ile** | **3,4,5** |
| **18417** | **synonymous** | **orf1ab** | **c.18153T>C** | **p.Asp6051Asp** | **4** |
| **19459** | **missense** | **orf1ab** | **c.19195A>G** | **p.Ile6399Val** | **1** |
| **19563** | **synonymous** | **orf1ab** | **c.19299G>A** | **p.Leu6433Leu** | **2** |
| **20262** | **synonymous** | **orf1ab** | **c.19998A>G** | **p.Leu6666Leu** | **3,4,5** |
| **20320** | **missense** | **orf1ab** | **c.20056C>T** | **p.His6686Tyr** | **3,4,5** |
| **21618** | **missense** | **S** | **c.56C>G** | **p.Thr19Arg** | **1,2,3,4,5** |
| **21987** | **missense** | **S** | **c.425G>A** | **p.Gly142Asp** | **1,2,3,4,5** |
| **22028** | **disruptive inframe deletion** | **S** | **c.467-472delAGTTCA** | **p.Glu156-Arg158delinsGly** | **1,2,3,4,5** |
| **22227** | **missense** | **S** | **c.665C>T** | **p.Ala222Val** | **1,2** |
| **22917** | **missense** | **S** | **c.1355T>G** | **p.Leu452Arg** | **1,2,3,4,5** |
| **22995** | **missense** | **S** | **c.1433C>A** | **p.Thr478Lys** | **1,2,3,4,5** |
| **23403** | **missense** | **S** | **c.1841A>G** | **p.Asp614Gly** | **1,2,3,4,5** |
| **23480** | **missense** | **S** | **c.1918T>C** | **p.Ser640Pro** | **3,4,5** |
| **23604** | **missense** | **S** | **c.2042C>G** | **p.Pro681Arg** | **1,2,3,4,5** |
| **24410** | **missense** | **S** | **c.2848G>A** | **p.Asp950Asn** | **1,2,3,4,5** |
| **24745** | **synonymous** | **S** | **c.3183C>T** | **p.Val1061Val** | **3,4,5** |
| **24872** | **missense** | **S** | **c.3310G>T** | **p.Val1104Leu** | **3,4,5** |
| **25469** | **missense** | **ORF3a** | **c.77C>T** | **p.Ser26Leu** | **1,2,3,4,5** |
| **25841** | **missense** | **ORF3a** | **c.449A>G** | **p.His150Arg** | **3,4,5** |
| **25855** | **missense** | **ORF3a** | **c.463G>T** | **p.Asp155Tyr** | **1,2** |
| **25889** | **missense** | **ORF3a** | **c.497C>T** | **p.Ser166Leu** | **3,4,5** |
| **25899** | **synonymous** | **ORF3a** | **c.507T>C** | **p.Ile169Ile** | **3,4,5** |
| **26767** | **missense** | **M** | **c.245T>C** | **p.Ile82Thr** | **1,2,3,4,5** |
| **27204** | **frameshift** | **ORF6** | **c.6delT** | **p.His3fs** | **3** |
| **27638** | **missense** | **OFR7a** | **c.245T>C** | **p.Val82Ala** | **1,2,3,4,5** |
| **27739** | **missense** | **OFR7a** | **c.346C>T** | **p.Leu116Phe** | **3,4,5** |
| **27752** | **missense** | **OFR7a** | **c.359C>T** | **p.Thr120Ile** | **1,2,3,4,5** |
| **28247** | **conservative inframe deletion** | **ORF8** | **c.355-360delGATTTC** | **p.Asp119-Phe120del** | **1,2,3,4,5** |
| **28270** | **intergenic** | **ORF8-N** | **n.28271delA** |  | **1,2,3,4,5** |
| **28461** | **missense** | **N** | **c.188A>G** | **p.Asp63Gly** | **1,2,3,4,5** |
| **28881** | **missense** | **N** | **c.608G>T** | **p.Arg203Met** | **1,2,3,4,5** |
| **29358** | **missense** | **N** | **c.1085C>T** | **p.Thr362Ile** | **3,4,5** |
| **29402** | **missense** | **N** | **c.1129G>T** | **p.Asp377Tyr** | **1,2,3,4,5** |
| **29427** | **missense** | **N** | **c.1154G>A** | **p.Arg385Lys** | **3,4,5** |
| **29631** | **missense** | **ORF10** | **c.74A>G** | **p.Asn25Ser** | **1,2** |
| **29742** | **intergenic** | **ORF10-END** | **n.29742G>T** |  | **1,3,5** |

**SNV: single nucleotide variant, CDS: coding sequence. ORF: open reading frame. The intergenic mutations didn’t cause any aminoacid changes.**
